# Transcatheter aortic valve durability: a contemporary clinical review

**DOI:** 10.3389/fcvm.2023.1195397

**Published:** 2023-05-09

**Authors:** Nicholas J. Montarello, Yannick Willemen, Gabriela Tirado-Conte, Alejandro Travieso, Gintautas Bieliauskas, Lars Sondergaard, Ole De Backer

**Affiliations:** The Heart Centre, Rigshospitalet, Copenhagen University Hospital, Copenhagen, Denmark

**Keywords:** transcatheter aortic valve implantatíon, transcatheter aortic valve, durability, structural valve degeneration (SVD), bioprosthetic valve failure

## Abstract

Encouraged by randomized controlled trials demonstrating non-inferiority of transfemoral transcatheter aortic valve implantation (TAVI) compared to surgical aortic valve replacement (SAVR) across all surgical risk categories, there has been a dramatic increase in the use of TAVI in a younger patient cohort with severe aortic stenosis, endorsed by both European and American Cardiac Societies. However, the standard use of TAVI in younger, less co-morbid patients with a longer life expectancy can only be supported if there is sound data demonstrating long-term durability of transcatheter aortic valves (TAVs). In this article, we have reviewed available randomized and observational registry clinical data pertaining to TAV long-term durability, placing emphasis on trials and registries using the new standardized definitions of bioprosthetic valve dysfunction (BVD) and bioprosthetic valve failure (BVF). Despite inherent difficulties in interpreting the available data, the determination reached is that the risk of structural valve deterioration (SVD) is potentially lower after TAVI than SAVR at 5 to 10 years, and that the two treatment modalities have a similar risk of BVF. This supports the adoption of TAVI in younger patients evident in current practice. However, the routine use of TAVI in younger patients with bicuspid aortic valve stenosis should be cautioned due to insufficient long-term TAV durability data in this particular patient population. Finally, we highlight the importance of future research into the unique potential mechanisms that can potentially contribute to TAV degeneration.

## Introduction

Transcatheter aortic valve replacement (TAVI) has become the therapeutic standard of care for selective cohorts of patients with severe symptomatic aortic stenosis (AS) across all surgical risk categories (1–6). The European Society of Cardiology (ESC) currently recommends that patients 75 years or older receive TAVI rather than surgical aortic valve replacement (SAVR) (7) while the American Heart Association (AHA) guidelines are more liberal, recommending as a Class 1 indication either transfemoral TAVI or SAVR for patients 65 years or older (8). However, the enthusiastic shift of TAVI utilization in younger, less co-morbid patients with longer life expectancy needs to be significantly tempered and influenced by an understanding of the durability of transcatheter aortic bioprosthesis. The difficulty is that, at present, there is a paucity of randomized controlled trial data regarding the long-term durability of transcatheter aortic valves (TAVs) with most of this data being derived from observational registry work. In this article, we review the available clinical data relating to long-term durability of TAVs which should be a major consideration when contemplating the routine adoption of TAVI in a younger patient population.

### Durability of surgical aortic valves: gold standard?

The adoption of new technologies and treatments are often dependent on comparative performance and outcome when measured against current accepted best practice. Transcatheter valve durability is typically compared to surgical bioprosthesis durability, readily accepted as the “gold standard”. But should it be? Single centre data in 2,659 patients assessing the long-term durability of surgical Carpentier-Edwards (Edwards LIfesciences, US) bovine pericardial prostheses indicates that structural valve deterioration (SVD) occurs in 21% of patients at 15 years and 51% at 20 years (9). Better results were reported in a cohort of 12,569 patients also treated with Carpentier-Edwards PERIMOUNT bovine pericardial valves, where the rate of re-operation was 1.9% and 15% at 10 and 20 years, respectively (10). Bovine pericardial valves have been shown to have superior haemodynamic profiles and late survival rates compared with porcine valves (11). However, a systematic review of 167 studies and 12 Food and Drug Administration (FDA) reports concluded that reporting bioprosthetic surgical valve durability in the literature is characterized by such variable definitions and inadequate long-term follow-up, that it makes the comparison between different types valves difficult (12). Fundamentally, the incidence of SVD is challenging to establish in the surgical literature because freedom from valve re-intervention is a frequent clinical end-point for diagnosing SVD (13). This underestimates its true incidence, as re-operation may not be proposed to poor surgical candidates, echocardiographic surveillance is often lacking in surgical patients, and some surgical patients may die before there is echocardiographic detection of SVD. Hence, surgical bioprosthesis durability, as currently determined, may not be the best benchmark comparator for TAV durability; all of which highlights the essential requirement of having a standardised definition of valve durability, including echocardiographic findings.

### Definition of bioprosthetic valve durability

#### Historical definition

Bioprosthetic valve dysfunction (BVD) has traditionally been divided into SVD and non-SVD. Structural valve deterioration refers to intrinsic degeneration or dysfunction of the prosthetic valve materials with the principal mediators including leaflet calcification, leaflet tear, stent fracture or stent creep, manifest as inward bending of a stent post. Non-SVD is defined as secondary processes associated with the valve such as patient prosthesis mismatch (PPM), paravalvular leak, pannus in-growth, valve thrombosis and endocarditis.

#### New standardised definition

A first standardised definition of bioprosthetic valve durability was provided in 2017 based on the consensus statement from the European Association of Percutaneous Cardiovascular Interventions (EAPCI), the ESC and the European Association for Cardio-Thoracic Surgery (EACTS) (14) (Table 1). Here, valve durability was divided in BVD and bioprosthetic valve failure (BVF). In 2021, the Valve Academic Research Consortium (VARC) 3 published an alternative definition of bioprosthetic valve durability that required permanent morphologic change of the bioprosthesis to be identified in addition to haemodynamic changes before SVD could be diagnosed (15, 16) (Table 1). This definition recognizes that haemodynamic valve deterioration may be caused by factors other than SVD and that utilizing only haemodynamic criteria may overestimate the incidence of true SVD. As a corollary, the failure to acknowledge the presence of early morphologic changes within the valve can underestimate the incidence of SVD.

**Table 1:** EAPCI/ESE/EACTS and VARC-3 standardised criteria of SVD.

Bioprosthetic valve dysfunction classified into 4 groups:	
Category 1	◊ Structural valve deterioration ○ Moderate ⇒ Mean gradient ≥20 mmHg; *or* ⇒ 3 months post-procedure • Increase in mean gradient ≥10 mmHg; *or* • Moderate intra-prosthetic AR ○ Severe ⇒ Mean gradient ≥40 mmHg; *or* ⇒ 3 months post-procedure • Increase in mean gradient ≥20 mmHg; *or* • Severe intra-prosthetic AR
Category 2	◊ Non-structural valve deterioration ○ Prosthesis-patient mismatch ⇒ Moderate prosthesis-patient mismatch • iEOA ≤0.85 cm²/m² (≥ 3 months post-procedure) ⇒ Severe prosthesis-patient mismatch • iEOA ≤0.65 cm²/m² (≥ 3 months post-procedure) ○ More than mild paravalvular leak
Category 3	◊ Bioprosthetic valve thrombosis ○ Thrombus on any prosthesis structure leading to dysfunction
Category 4	◊ Infective endocarditis ○ Diagnosed according to modified Duke criteria
Bioprosthetic valve failure defined as one of following 3 criteria:	
1. Valve-related death; or	
2. Severe hemodynamic structural valve deterioration; or	
3. Aortic valve-reintervention following diagnosis of bioprosthetic valve dysfunction	
VARC-3 standardised criteria of SVD.	
Stage 1	Early morphological changes without hemodynamic changes
Stage 2	◊ Hemodynamic changes (assessed 1 to 3 months post-procedure) ○ Increase in mean gradient ≥10 mmHg resulting in mean gradient ≥20 mmHg with concomitant decrease in EOA ≥0.3 cm² *or* ≥25%, *and/or* decrease in Doppler velocity index ≥0.1 *or* ≥20% ◊ Intra-prosthetic regurgitation ○ New occurrence, *or* increase of ≥1 regurgitant grade(s) resulting in ≥moderate AR
Stage 3	◊ Hemodynamic changes (assessed 1 to 3 months post-procedure) ○ Increase in mean gradient ≥20 mmHg resulting in mean gradient ≥ 30 mmHg with concomitant decrease in EOA ≥0.3 cm² *or* ≥25%, *and/or* decrease in Doppler velocity index ≥0.1 *or* ≥20% ◊ Intra-prosthetic regurgitation ○ New occurrence, *or* increase of ≥2 regurgitant grades resulting in severe AR

AR, aortic regurgitation; EOA, effective orifice area; iEOA, indexed effective orifice area; SVD, structural valve deterioration.

Not surprisingly, given the relatively recent standardised definition of valve durability, long-term data regarding BVD are still conflicting and are accessible for first-generation devices only owing to the shorter follow-up available of latest generation devices.

### Transcatheter aortic bioprosthesis: five-year freedom from SVD

In the past few years, the outcomes of a number of TAVI studies and registries evaluating mid-term TAV durability have been published.

Randomized trials include the PARTNER, CoreValve US Pivotal, SURTAVI-IR and NOTION trials. The PARTNER-1 trial demonstrated no evidence of SVD at 5-year follow-up (17, 18). Further, the PARTNER-1A sub-study showed comparable echocardiographic performance of TAVs and surgical aortic valves, with a mean transvalvular gradient of 10.7 mmHg and 10.6 mmHg, and an aortic valve area of 1.6 cm^2^ and 1.5 cm², respectively (17, 19). This attested to the acceptable haemodynamic profile of TAVs up to 5 years post-implantation even though moderate or severe paravalvular regurgitation, not incorporated in the definition of SVD, was more prevalent in the TAVI group. More recently, using standardised definitions of valve durability (14–16), pooled data from the CoreValve US High Risk Pivotal (20) and SURTAVI-IR (4) randomised clinical studies showed a significantly lower rate of BVD with TAVI utilizing a self-expanding TAV [CoreValve 88% and Evolut-R 12% (Medtronic, US)] compared with SAVR through 5 years (7.8% vs. 14.2%, HR 0.50, *p* < 0.001) (21). This was driven by a reduced 5-year incidence of SVD of 2.2% in the TAVI cohort vs. 4.4% in the SAVR cohort (HR 0.46, *p* < 0.004) and a reduced 30-day discharge incidence of severe PPM in TAVI treated patients of 3.7% compared to 11.8% in patients undergoing SAVR (HR 0.29, *p* < 0.001). Of clinical importance, the development of BVD across the different treatment modalities imparted a 1.5-fold higher risk for all-cause mortality (*p* = 0.004), cardiovascular mortality (*p* < 0.001) and hospitalization for valve disease or worsening heart failure (*p* = 0.001) at 5 years. In the Nordic Aortic Valve Intervention (NOTION) trial, 280 patients were randomized to either TAVI with CoreValve (*n* = 145) or SAVR (*n* = 135) (22). The mean age was 79.1+/- 4.8 years and the mean STS predicted risk of mortality score was 3 +/- 1.7%, indicative of a lower risk patient cohort. At five years, the TAVI cohort had a larger prosthetic valve area (1.7 vs. 1.2 cm^2^, *p* < 0.001) with a corresponding lower mean trans-prosthetic gradient (8.2 vs. 13.7 mmHg, *p* < 0.001) than the SAVR cohort. However, transcatheter treated patients had increased incidence of moderate and severe paravalvular aortic regurgitation (8.2 vs. 0%, *p* < 0.001).

The largest 5-year mid-term bioprosthetic TAV durability registry data is obtained from the FRANCE-2 Registry (23). This registry comprised 4201 patients undertaking TAVI with self-expanding (SE, 32%) or balloon-expandable (BE, 68%) TAVs and revealed a rate of severe and moderate/severe SVD of 2.5% and 13.3%, respectively, at 5 years from the procedure in surviving patients. Of note, the 5-year rate of moderate and severe SVD was 8.9% and 0% for SE device, and 13.8% and 4.1% for BE TAVs. The occurrence of severe SVD was not correlated with excess mortality, possibly due to the fact that the majority of severe SVD cases were defined by an increased mean gradient instead of severe aortic regurgitation.

### Transcatheter aortic bioprosthesis durability: data beyond 5 years

There is limited data pertaining to the long-term durability of TAVs predominantly due to their initial use in older and higher risk patients that often did not survive beyond 7 to 8 years (24). The NOTION trial is therefore particularly significant in that it provides randomized data beyond 5 years and exclusively evaluates TAV durability in a younger and, more importantly, lower risk patient cohort with a longer life expectancy. Jørgensen et al. recently reported the 8-year outcomes for patients enrolled in this trial (25). The results represent the longest reported follow-up of a patient population randomised to TAVI or SAVR and demonstrated that there was a significantly lower rate of SVD in the TAVI group compared to SAVR (13.9% vs. 28.3%, *p* = 0.0017), but a similar risk of BVF (8.7% vs. 10.5%, *p* = 0.61). The risk of severe SVD was 2.2% in the TAVI cohort vs. 6.8% in the SAVR cohort, *p* = 0.068). No patient experienced clinical valve thrombosis, whilst the cumulative frequency of endocarditis was 7.2% and 7.4% for patients treated with TAVI and SAVR, respectively. Importantly, TAVI patients had a greater effective orifice area and lower transvalvular gradient at every yearly follow-up when compared to patients managed with SAVR.

**Figure 1:**
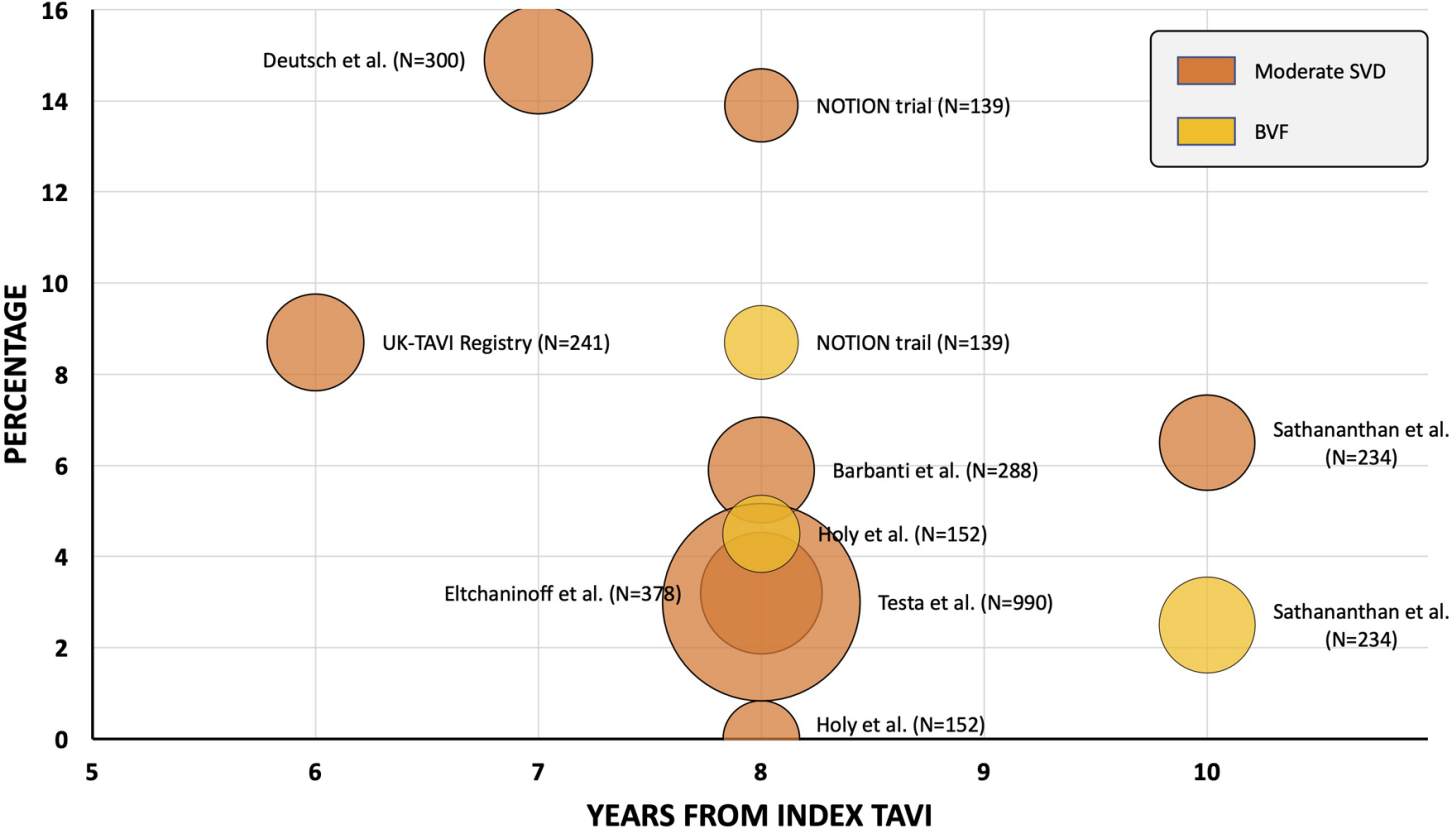
Transcatheter aortic valve freedom from moderate structural valve deterioration and bioprosthetic valve failure data—beyond 5 years. Orange = moderate structural valve deterioration; yellow = bioprosthetic valve failure. Bubble chart representative of study cohort size.TAV, transcatheter aortic valve; TAVI, transcatheter aortic valve replacement.

Following the establishment of the EAPCI/ESE/EACTS standardised criteria of SVD, a growing number of trials and registries have reported outcomes utilizing this haemodynamically based definition after TAVI with SAPIEN (Edwards Lifesciences, US) or CoreValve for up to 7 and 8 years (Figure 1). Deutsch et al. described late-outcomes and SVD in 300 patients managed with TAVI (71% SE and 29% BE) (26). Following a median follow-up of 7.14 years, the true incidence of SVD was significantly lower in the SE group compared to the BE cohort (11.8% vs. 22.6%, *p* = 0.01). Barbanti et al. reported on a cohort of 288 patients treated with CoreValve (82.3%) and SAPIEN XT (16.7%) and established an 8-year cumulative rate of moderate and severe SVD of 5.9% and 2.4%, respectively (27). Eltchaninoff et al. showed, in a cohort or 378 patients treated with BE valves, that there was an incidence of SVD and BVF at 8-year follow-up of 3.2% and 0.6%, respectively (28). Holy et al. reported long-term results of 152 successive patients who had proceeded to TAVI with CoreValve between 2007 and 2011 (29). Echocardiographic follow-up was performed at 6.3 +/- 1.0 years in 88% of patients surviving beyond 5 years. No case of SVD was recorded and 5 patients (3.3%) had undergone redo-TAVI or surgery due to paravalvular leak. Testa et al. reported on 990 patients undergoing TAVI with CoreValve/Evolut-R and documented an 8-year cumulative incidence of moderate SVD and severe SVD of 3.0% and 1.6%, respectively (30). Sathananthan et al. reported on 234 consecutive patients treated with SAPIEN (77.4%), Cribier-Edwards (Edwards Lifesciences, US) (20.9%) or CoreValve (1.7%) and reported a 10-year cumulative incidence of SVD and BVF of 6.5% and 2.5%, respectively (31). In addition, the UK TAVI Registry evaluated the incidence of SVD in 241 patients treated with SE (66%) and BE (34%) TAVs with a follow-up period ranging from 5 to 10 years (median follow-up 5.8 years) (32). In this registry, the reported frequency of moderate SVD and severe SVD was 8.7% and 0.4%, respectively. There was no difference in the rate of moderate SVD between the SE or BE devices. Only 1 reported case of severe SVD was seen and occurred in the SE cohort.

## Discussion

The utilisation of TAVI has expanded dramatically over the preceding decade. Although mid-term follow-up studies exhibit favourable outcomes following transfemoral TAVI, very limited long-term TAV durability data exist. Despite this, the most recent AHA guidelines state that “*for symptomatic patients with severe aortic stenosis who are 65 to 80 years of age and have no anatomic contraindication to transfemoral TAVI, either SAVR or transfemoral TAVI is recommended after shared decision-making about the balance between expected patient longevity and valve durability”* (Class 1, Level of Evidence A). Unfortunately, what complicates matters for the treating physician is that the availability and interpretation of long-term TAV durability data upon which to base decision-making is problematic for a number of reasons (33). Firstly, little TAV durability data exists beyond 10 years. Secondly, TAVI is routinely utilised in co-morbid elderly patients who may die from non-cardiac causes and, consequently, SVD may go unobserved in many TAVI trials. Thirdly, annual surveillance echocardiography is more frequently performed post-TAVI than post-SAVR (34). As such, non-clinically significant SVD may be far more commonly detected long-term in TAVI vs. SAVR which makes a comparison between the two more difficult. Fourthly, the incongruous definitions of SVD utilized in trials and registries leads to uncertainty about the true incidence of SVD following TAVI. Finally, there have been iterative improvements in pre-procedural planning, stent technology, implantation technique and operator experience which is anticipated to improve long-term durability for more recently implanted, newer generation valves. As an example, using the VARC-3 definition of SVD, a recent trial described that the second-generation BE SAPIEN XT valve had an increased likelihood of SVD compared with the third-generation SAPIEN 3 TAV, which had a comparable incidence of SVD compared to surgical bioprosthesis (15, 16).

Despite these inherent difficulties, there is good early data—using the new standardized EAPCI/ESC/EACTS criteria—that the risk of SVD is potentially lower after TAVI than SAVR at 5 to 10 years. This, together with well-documented improved valve effective orifice area and lower transvalvular gradients following TAVI compared to SAVR is encouraging and lends support for the expansion of TAVI to patients with a longer life expectancy. However, there is one major caveat. With the progressive expansion of TAVI towards younger patients, Heart Teams are increasingly encountering patients with severe bicuspid aortic valve (BAV) stenosis. These patients were excluded from the large randomized TAVI trials (17, 18, 20, 22).This, together with the absence of any TAV durability data in BAV beyond 2 years should caution against the use of TAVI as a first line therapy for patients with severe AS and BAV anatomy.

The limited durability data beyond 5 years comparing SE and BE devices does not allow sufficient distinction to be made to influence clinical practice. These devices have only been directly compared in a small number of mid-term randomized controlled trials with discordant results (35, 36). Available registry data suggests a lower rate of SVD in SE devices (26) with a recent propensity-matched analysis of patients undergoing TAVI with small aortic annuli demonstrating increased SVD in BE valves driven by increased PPM in patients treated with BE valves (37).

Clearly, future research focus on the potential mechanism of TAV degeneration is needed, so that long-term TAV durability can be improved. Whilst it is recognised that TAVs can degenerate in a manner similar to surgical bioprosthesis, durability of TAVs may be impacted as a result of the potential trauma arising due to initial valve preparation and balloon dilatation or as a result of suboptimal leaflet coaptation, leaflet pin-wheeling or asymmetric stent frame expansion (38). Additionally, prosthetic valve factors including BE vs. SE platforms, supra-annular vs. intra-annular leaflet position, length of leaflet coaptation, and the ability to achieve commissural alignment may be important. This all needs to be further studied, as does the role of anti-thrombotic pharmacotherapy in preventing TAV leaflet thickening and its potential impact on future SVD.

## Conclusion

Available randomized and registry observational data using new standardized definitions of SVD support the use of TAVI in younger patients with severe, symptomatic AS, recommended by recently updated Societal Guidelines. The risk of SVD is potentially lower at 5 to 10 years following TAVI compared to SAVR, with both treatment modalities displaying a similar risk of BVF. However, restraint should be exercised when treating young patients with a bicuspid aortic valve stenosis due to insufficient long-term TAV durability data in these patients.
